# Motor and Sensory Dysfunction in Musician’s Dystonia

**DOI:** 10.2174/157015913804999531

**Published:** 2013-01

**Authors:** Florence C F Chang, Steven J Frucht

**Affiliations:** Mount Sinai Medical Center, 5 East 98^th^ Street, Box 1138, First Floor, New York, NY 10029

**Keywords:** Botulinum toxin, cortical plasticity, dystonia, musician, rehabilitation, trihexyphenidyl.

## Abstract

Musicians’ dystonia is a task-specific and painless loss of motor control in a previously well-executed task. It is increasingly recognized in the medical and musical community. Recent advances in neuroimaging, transcranial magnetic stimulation and novel techniques in electroencephalography have shed light on its underlying pathophysiology. To date, a deranged cortical plasticity leading to abnormal sensorimotor integration, combined with reduced inhibition across several levels of the motor pathway are likely mechanisms.This paper reviews the various phenomenology of musician’s dystonia across keyboard, string, brass, flute and drum players. Treatment is often challenging. Medical therapies like botulinum toxin injection and rehabilitation method with sensorimotor training offer symptomatic relief and return to baseline performance to some musicians.

## INTRODUCTION

Musician’s dystonia (MD) is a focal, task-specific, painless disorder of motor control, often resulting in the termination of professional performance. It affects as many as 1 in 200 musicians during their career [1] and 1-14% of musicians seen in a performing arts medical center [2,3]. These numbers may be underestimated, and the diagnosis of musician’s dystonia is often delayed. The first record of musician’s dystonia dates back to 1830 from Robert Schumann’s diaries. Subsequent descriptions by Gowers on focal hand dystonia in musicians were later erroneously attributed to a psychogenic cause. It was not until 1982 with Marsden and Sheehy classic description of writer’s cramp, a type of focal task-specific dystonia, that an organic etiology became widely accepted. Recent publicity surrounding the poignant story of Leon Fleischer and his return to two-handed playing has brought this condition to the public’s and medical professional’s attention. Musician’s dystonia has been described to affect the hand and embouchure of virtually every instrument, to affect professionals and amateurs, and to occur in classical and traditional musicians throughout the world. 

Over the last decade, investigators have improved our understanding of the underlying pathophysiology and provided the basis for medical treatment and development of specific rehabilitation programs. Advances in neuroimaging and transcranial magnetic stimulation on both healthy musicians and musicians with dystonia have supported the concept of aberrant cerebral plasticity in the disorder. A review by Altenmuller in 2003 suggested musician’s cramp occurred secondary to overuse of a specific motor skill, leading to degradation in motor memory and subsequent loss of voluntary control of motor tasks [3]. This is supported by a study of trained monkeys by Byl, who demonstrated that overuse can lead to abnormal cortical representation of somatosensory receptive fields [4]. Conti (2008) reviewed a total of 960 musicians with focal dystonia and characterized the phenomenology and pattern of involvement in each class of musical instruments. The pattern of involvement seems related to the specific technical demand of the musical instruments on the hand and the individual digits that were involved [5]. Jankovic (2008) published a review of movement disorders in musicians, specifically musician’s focal dystonia, with the aim to raise the musical and medical community’s awareness of the condition [6]. A similar publication (2009) highlighted sensory rehabilitation and botulinum toxin as effective symptomatic relief [7]. Altenmuller in the same year suggested that psychological factors contributed to musician’s hand dystonia, and presented psychological findings in a study of healthy musicians, musicians with chronic pain and those with musician’s dystonia [8]. The same author published his latest review in 2010, outlining the pathophysiology, phenomenology, triggering factors and treatment options available to date [9].

Since 2010, there have been numerous discoveries germane to the pathophysiology and treatment of the disorder using a range of techniques. Functional magnetic resolution imaging studies (fMRI) on embouchure dystonia patients and guitarists with focal hand dystonia found increased sensorimotor cortex activation [10] and reduced thalamic and basal ganglia activation. In addition, an fMRI study by Granert found an association with larger middle putaminal volume in patients with musician’s dystonia compared with healthy musicians [11]. Using electroencephalography (EEG) oscillatory technique, Ruiz found an association between MD and EEG error detection phase synchronization in 6-8Hz frequency band during a musical performance [12]. In 2012, Bradley found reduced sensitivity in temporal discrimination thresholds which was comparable to other adult onset primary dystonia subjects. There have been further associations between anxiety and perfectionistic traits as possible endophenotypes of musicians’ dystonia. Enders suggested that anxiety and musician’s dystonia may share a common pathophysiological mechanism, rather than being a secondary reaction to dystonia [13]. Schmidt raised the intriguing possibility of a genetic contribution to MD, finding nearly half of 116 musician dystonia subjects had one or more additional features of primary dystonia [14], however MD patients with a family history did not test positive for mutations known to cause dystonia in a large case series [15]. This suggests perhaps a yet to be discovered gene may be responsible for MD subjects with a positive family history. 

Advancements in therapy have mainly been in the field of rehabilitation. Improvement in motor control occurred after constraint-induced therapy, and sustained improvements after sensorimotor retraining were reported [16, 17]. In contrast, cathodal transcranial direct current simulation failed to improve motor control in subjects with musician’s dystonia [18].

## RISK FACTORS

The patients affected by musician’s dystonia were predominantly male [19] with a mean age of onset at 35, and age of symptom onset ranging between 16 to 75 years of age [5].

There are observations to suggest that a combination of genetic and environmental influences produce the clinical picture of musician’s dystonia. A social and cultural change in classical music occurred in the nineteenth century, with virtuosos performing in concert halls with increasingly demanding repertoire [20]. MD is more commonly seen in professional classical musicians than jazz musicians or amateur musicians [21]. The needs of the classical musician to play with near-perfect accuracy, and the demands of practice are thought to generate a breakdown of normal somatosensory representation in the cortex. A change in practice technique or increased duration was noted as precipitating events leading to MD in one study of 18 of 58 patients [2]. Playing an instrument with higher spatial sensorimotor demand, such as the keyboard, high string, guitar or brass instruments is associated with a higher rate of musician’s dystonia [22]. Other factors such as being a soloist or a perfectionistic or controlling personality were found to also be risk factors for musician’s dystonia [3, 9, 13]. There have been case reports of peripheral trauma or neuropathy triggering focal task specific dystonia in musicians. Ulnar neuropathy has been found in up to 40% of a case series of 73 musicians with hand dystonia [23]. The worsening of ulnar neuropathy led to increased severity of dystonia whereas an improvement in neuropathy resulted in resolution of dystonia [23]. Ulnar neuropathy can produce similar peripheral neurophysiological findings to those with focal hand dystonia but without increased cortical excitation. For example, co-contraction of agonist and antagonist muscles are associated with reduced reciprocal inhibition of H reflex and decreased spinal inhibition. However, those ulnar neuropathy does not have increased cortical excitation seen in patients with focal task-specific dystonia [23].

There is evidence to suggest an endophenotype with a predilection to the development of dystonia. An underlying unknown genetic cause is suggested by a study whereby 36 of 185 patients with musician’s dystonia had a family history of task-specific dystonia, yet the patients with a positive family history screened negative for DYT1 and THAP1 mutations, except for 2 patients with early onset MD [15]. In a medical clinic for performance artists, 9% of patients with musician’s dystonia had a family history of focal task-specific dystonia [1]. Findings of cortical excitability and impaired sensorimotor integration in the cerebral hemisphere responsible for the unaffected hand of musicians with dystonia suggests an endophenotype that has a predisposition towards the development of dystonia [24, 25]. In addition, spread of dystonia to the opposite hand in musician’s dystonia and writer’s cramp further supports the concept of an underlying endophenotype [5].

## NEUROPHYSIOLOGY IN HEALTHY MUSICIANS

In order to understand the underlying cause of musician’s dystonia, it is important to appreciate how healthy musicians differ from healthy controls. Healthy musicians have enhanced cortical activation associated with reduced cortical inhibition compared to non-musicians.Neurophysiological findings in transcranial magnetic stimulation (TMS) have supported a more focused motor cortical activation in addition to the prevention of unwanted spread of activation in healthy musicians compared to non-musicians [26]. Healthy musicians have increased slope for both corticospinal and intracortical input-output relationships compared to non-musicians. In additions, healthy musicians were found to have a more rapid increase in primary motor cortex activation than non-musicians during a novel tapping task [27]. Healthy guitarists and keyboard musicians have reduced interhemispheric inhibition in a TMS study [28]. These studies may explain the superior fine motor control and performance in musicians compared with healthy controls. 

There is evidence of cortical plasticity in both healthy non-musicians and musicians. The practice of music involves an acquisition of new motor skill through repetitions. Repetitive motor tasks are associated with a reduction in cortical inhibition and increased cortical excitability in healthy subjects [29]. Repetitive practice of complex finger movement increases activity in the supplementary motor and premotor areas and cortical representation of fingers in primary motor cortex. This effect diminishes back to baseline after one week [30]. Using magnetoencephalography, Elbert found enlarged cortical representations corresponding to second, third, fourth and fifth digit of the left hand in professional violinists [31]. There is also evidence to suggest that musicians have increased size of cortical motor pathway volume compared to healthy non-musicians. The mean cerebellar volume is greater in male musicians than male non-musicians [32]. In keyboard musicians, there is a negative correlation between the time of musical training and the size of the hand motor areas and anterior corpus callosum measured by MRI, compared to non-musicians [33]. This suggests that early musical training is associated with a larger primary hand motor area and anterior corpus callosum, which contains fibers from the motor and supplementary motor areas.

## PATHOPHYSIOLOGY

There are findings to support loss of inhibition, excessive cortical plasticity, altered sensory processing and impaired sensorimotor integration contributing to the pathophysiology of musician’s dystonia. 

## LOSS OF INHIBITION

Most forms of dystonia have been associated with a reduction in inhibition. One form of dystonia, writer’s cramp, is a task-specific dystonia similar to musician’s dystonia. It is associated with reduction of inhibition which occurs in multiple levels: the motor cortex, subcortical and spinal levels. Using magnetic resonance spectroscopy, a significant decrease in gamma-aminobutyric acid (GABA) levels in the sensorimotor cortex and lentiform nuclei of patients with writer’s cramp was found compared to controls [34]. Reduction in GABA correlates with reduced intracortical inhibition found in TMS studies and contingent negative variation techniques [35] in patients with writer’s cramp [25]. Abnormal increases in cortical excitability and reduction of cortical silence period generated by attenuated intracortical inhibitory circuits may play a role in maladaptive plasticity during repetitive hand movements [36]. Functional MRI studies found bilateral increased signal in the putamen, caudate nucleus, internal globus pallidus and lateral thalamus in patients with writer’s cramp relative to controls during a task-related activity [37]. This supports the concept of impaired center-surround inhibition within basal ganglia-thalamic circuits leading to excess activation of the sensorimotor cortex, producing dystonia. At the spinal level, reciprocal inhibition between agonist and antagonist muscles is reduced in patients with focal hand dystonia. This can either lead to altered input to the spinal cord or abnormal control of spinal interneurons mediating presynaptic inhibition within the spinal cord [38]. This results in abnormal co-contraction of agonist and antagonist muscles with overflow of inappropriate muscle activation observed in EMG studies [39].

Similar reduction in cortical inhibition is shown in patients with musician’s dystonia. Using TMS, musicians with dystonia have loss of cerebellar to cortical inhibition, compared to healthy controls [40]. The reduction of cortical inhibition is associated with and probably facilitates the acquisition of new motor skills. However, it is uncertain what causes the pathological reduction in inhibition leading to the clinical presentation of musician’s dystonia.

## ALTERED SENSORY PROCESSING

One of the possible mechanisms causing excessive motor output is thought to be decreased inhibition or increased sensory excitation. Several neurophysiology and TMS studies support this finding. Paired associative stimulation using TMS transiently induces an abnormal increase in excitability in the primary somatosensory cortex. In addition, intracortical inhibition is increased in the same area [41]. There is also an increase in cortical response during auditory evoked potentials in patients with musician’s dystonia compared to controls [42]. Patients with focal task-specific dystonia have reduced perception of tonic vibration [43] and decreased activation of the contralateral supplementary motor and somatosensory cortex on vibration of the affected and unaffected hand [44]. The degree of impairment correlated with the severity of the dystonia. Interestingly, application of vibration to hand muscles strongly reduced the short interval cortical inhibition in all hand muscles irrespective of spatial organization in musician’s dystonia patients [45]. In contrast, the same vibration had lesser effect on cortical excitability in patients with writer’s cramp. Perhaps sensory information has a greater role in the causation of pathological changes in musician’s dystonia than writer’s cramp.

In addition, both task-specific dystonia and musicians with focal dystonia have abnormal temporal discrimination threshold compared to controls [46]. Patients with focal task-specific dystonia have similar findings [47, 48]. This is thought to reflect abnormal temporal processing secondary to basal ganglia dysfunction, perhaps due to an imbalance of the activity within the globus pallidus interna [49].

Patients with musician’s dystonia and focal task-specific dystonia have disorganization of sensory representation on neuroimaging and magnetoencephalography (MEG). Musicians with focal dystonia without chronic pain have fusion of digital representation in the somatosensory cortex by MEG study [50]. Embouchure dystonia patients have an overlap in the cortical representation of the lip and tongue, with shifting of digit representation towards the lip representation zone on MEG study compared with controls [51]. Using functional MRI, similar disorganization of sensory representation was found in the sensory cortex of musicians with dystonia [52]. Patients with focal task-specific dystonia have similar findings using MEG and functional MRI [48, 53]. One study also found altered somatotopic representation in the putamen, which may contribute to the loss of functional selectivity of muscle activity [54]. Having a larger and overlapping cortical representation for a given body part may explain the finding of abnormal spatial discrimination in patients with focal hand dystonia [48]. An animal study using trained monkeys suggests that overuse can lead to abnormal sensory representation. It showed that repetitive stereotypic movements in a learning context led to a degradation of cortical representation of somatosensory information [4]. Simultaneous co-stimulation of cutaneous and kinesthetic afferents that are normally differentiated may explain the degradation of cortical representation as the remodeling of the hand map in the cerebral cortex is input-timing dependent [55].

## IMPAIRED SENSORY MOTOR INTEGRATION

Healthy pianists show reduction in motor and premotor cortex activation compared to healthy controls on functional MRI studies, [56] probably secondary to a more focused activation of motor control [26]. A study using EEG and a Go/No go paradigm found that musicians with dystonia have smaller movement related cortical potentials over the sensorimotor areas compared with healthy musicians. The finding was interpreted as reduced phase coupling in the non-retrieval motor program within the supplementary motor, premotor and sensorimotor area in musician’s with dystonia [57]. Similarly, there is a finding of diminished Bereitschaftpotential, a premotor movement potential, in writer’s cramp using the EEG technique [58]. Patients with musician’s dystonia have a further reduction of motor cortex activation and increased somatosensory cortex activation compared to healthy pianists on several functional MRI studies [10, 52, 59]. These were done on guitarists, keyboard and brass players with musician’s dystonia. Focal task-specific dystonia patients have similar findings on functional MRI [44]. The increased somatosensory cortex activation is in keeping with findings of a larger spatial integration of proprioceptive input in the hand cortex of musician’s dystonia subjects compared to healthy musicians [60]. In addition, it may be related to reduced intracortical inhibition or increased excitability as demonstrated by several TMS studies. Why this would lead to bilateral reduction in motor cortex activation [52] is uncertain. It may be explained by one study which found an impairment of focused motor cortical activation by vibratory input to a dystonia affected muscle [61]. Rosenkranz proposed that it may be secondary to either abnormal sensorimotor integration as an adaption to dystonic movements [26], or cortical reorganization. One case report alludes to a possible therapy utilizing the abnormal sensorimotor integration. One patient with embouchure dystonia found temporary relief of dystonic symptoms by cooling the affected body part [62]. It is possible that alteration of sensory feedback or muscle spindle activity by cooling led to a temporary counteracting or relief of abnormal cortical activation.

## PHENOMENOLOGY

Musician dystonia patients present with either a gradual or sudden deterioration in voluntary control of complex sensorimotor skills. For example, difficulty in control playing fast passages, fingers curling, lack of precision, irregularity of trills in keyboard and string players and lack of control in certain registers for brass or woodwind players with embouchure dystonia. These often arise in movements that are frequently practiced and often affect the hand that is subjected to the most technical demand in an instrument [9, 63]. For example, the right hand is more frequently affected in keyboard and plectrum instrumentalist, and the left hand in string players. Once the symptoms establish, the pattern of dystonic movements rarely change [5] and may worsen if playing a second instrument similar to the first [64]. Bilateral hand dystonia occurs in 5% of musician’s dystonia, unlike writer’s cramp where up to a third of patients experience spread of dystonia to the opposite hand [65].

## KEYBOARD MUSICIANS

As previously mentioned, the right hand is preferentially affected in keyboard players with musician’s dystonia. It affected up to 77% in one large review of 106 keyboard musicians with MD. The three most common patterns are: a combination of digit 4 and 5 (33%); isolated digit 4 (19%); isolated digit 2 (12%) [5]. This could be explained by the fact that the right hand digit 4 and 5 has the greater technical demand by carrying the melody line in keyboard music. The commonest involuntary movement is abnormal flexion of a digit, affecting up to 54% of keyboard players [5].

## PLUCKED INSTRUMENT MUSICIANS

Similar to keyboard musicians, the right hand is preferentially affected. Flexion movement was the commonest dystonic movement, most frequently affecting digits 3,4 and 5. This occurs either in combination or in isolation, and abnormal flexion movement occurs in 76% of patients [5]. This is the pattern seen in guitarists, whereas dystonia in banjo players mainly affect the thumb and index finger, reflecting the difference in technical demand between the two instruments.

## BOWED STRING PLAYERS

The left hand is more commonly affected, in 68% of string players in a large review of focal hand dystonia [5]. Again, flexion dystonic movements were most common, affecting 49% of players. Other dystonic movement pattern was extension (13%) and both flexion and extension (8%). It affected the left digit 4 and 5 in the majority of cases. Other digit patterns in order of declining frequency are: isolated digit 3 (18%); combination digit 3,4 and 5 (14%) and digits 3 and 4 (11%). The bow arm is less commonly involved but may carry a poorer prognosis. In a case series of string musicians with dystonia, none of the 5 patients with bowed arm dystonia returned to professional playing despite treatment [66].

## BRASS PLAYERS

Musician’s dystonia affects either the hand or the embouchure of brass players. From a large review of published cases of focal hand dystonia in musicians, either hand is equally affected in brass players. The most common digit affected in brass players is digit 3 (25%) or a combination of digits 2 and 3 (25% of a case series of 8 patients) [5]. Embouchure dystonia is painless, often triggered by certain musical practice techniques, and rarely by trauma. It is remarkably task-specific and occurs often in one instrument register or during certain styles of playing. From the principle author’s experience and observation of 89 patients with embouchure dystonia, there are several phenomenologic forms. These are associated with the register of the brass instrument. For example, the lip-locking phenotype tends to occur in low-register brass instruments like trombone and tuba, whereas the lip-pulling phenomenology occurred in high-register brass players. Lip tremor has a very high frequency, often occurring at the start of playing and only in one register. Videotaping and slow motion playback can be useful to differentiate the lip tremor from other phenotypes. Initially the dystonia is pitch specific, but it can spread to affect a perfect fourth or an octave in 69% of patients [67]. Over time, the dystonia can involve neighboring pitches. Interestingly, 6% of patients also had writer’s cramp, which often preceded the development of embouchure dystonia. This suggests an underlying endophenotype. As many as one in four patients can experience dystonia during other oral tasks [68].

## OTHER INSTRUMENTS

Dystonia affecting woodwind players may present like Meige syndrome or may even affect the jaw or tongue. Meige phenotypes present with involuntary movement of the upper and lower facial muscles during woodwind instrument playing. The jaw phenotype had involuntary jaw closure or lateral jaw movement during performance. The tongue phenotype had tongue incoordination triggered by passages that required tongue movement. Irregular intermittent tremors can affect those with the jaw phenotype [67].

Similar to brass players, either hand was equally affected in woodwind players, However, in a large review by Conti, the left hand is predominantly affected in 81% of flutists [5] and may be the hand with the greatest technical burden as the thumb of the left hand is used to support the instrument and negotiate the key. The commonest affected digit in woodwind players are a combination of digit 4 and 5 (24%); combination of 3,4 and 5 (18%) and isolated digit 3 (13%). It can manifest as either involuntary extension or flexion (41-46%) movement of the digit. 

Percussionist dystonia also equally affects either hand and abnormal flexion involving the wrist and forearm predominates [69]. Ulnar deviation of the wrist and tremor was occasionally reported. In tabla drum players, extension of the fingers of the dominant hand representing overflow dystonia, whilst performing rapid alternating movement of the left hand, was reported in 2 patients [70].

Laryngeal adductor dystonia is the predominant form of spasmodic dysphonia described in professional singers [71]. It manifests as mid-range loss of singing voice due to strain, roughness and loss of vibrato.

## TREATMENT OPTIONS

The treatment of musician’s dystonia is a definite challenge. Dystonic symptoms may be alleviated by medical therapy, botulinum toxin injection, ergonomic changes or rehabilitation [72]. Dystonic symptoms rarely resolve completely and in combination with treatment side effects, patients rarely return to professional playing. In addition, the highly demanding fine motor skills required for a professional music career requires an improvement in motor function close to baseline. Medical therapy or botulinum toxin injections were not found to be useful in patients with embouchure dystonia [73]. Medical therapies include anticholinergic agents like trihexyphenidyl and other medications like baclofen, primidone or phenytoin are occasionally helpful [72]. One third of 69 patients in a musician’s medicine clinic had subjective improvement on trihexyphenidyl, but experienced frequent side effects that made continuation of therapy intolerable [72].

Botulinum toxin injection is better tolerated than trihexyphenidyl. 49-69% of patients with musician’s dystonia of the hand found improvement in symptoms, and 36% had beneficial effects lasting after EMG guided botulinum toxin injection [72, 73]. The best outcome occurs when the muscles controlling the primary dystonic movements are injected, rather than compensatory movements [22]. It is especially useful for musicians with finger flexion dystonia [1]. The side effect of weakness limits the quantity and selection of muscles for injection. One study recommended injection in the hand muscles of musicians who had limited demand on lateral finger movement, such as guitarists and woodwind players. Keyboard players with a repertoire that does not require a large hand span or lateral finger movement can also benefit from botulinum toxin injection [72].

The concept of rehabilitation therapy is based on the hypothesis that musician’s dystonia is due to maladaptive plasticity resulting from aberrant learning of a motor task. The aim of the rehabilitation is to correct the abnormal somatotopic hand representation through a multidisciplinary team of a psychologist, occupational therapist, physical therapist and neurologist. Case reports and small case series of methods such as immobilization, constraint-induced training, proprioceptive and sensorimotor retraining had promising success. Constraint-induced training is a method of splinting the dystonic finger during instrumental practice. When motor control improved, the splints were gradually removed. Candia reported 8 out of 11 musicians experienced improvement in motor skills and somatosensory relationship of individual fingers in the affected cerebral cortex after 1 week of daily training, compared to the unaffected cerebral cortex [74]. Berque found a reduction in the frequency of abnormal movement after 8 months of constraint-induced therapy in 8 musicians with hand dystonia. Rosenkranz found that with 15 minutes of proprioceptive training, the abnormal sensorimotor organization seen in the dystonic subjects returned to a pattern seen in healthy musicians. In addition, objective measures of motor control and behavioral improvement correlated with improvement in sensorimotor organization [60]. Alternatively, sensorimotor training where subjects were given a gradual exposure to increasingly complex exercises led to an improvement in motor control. After sensorimotor training, the longest maintenance of improvement back to baseline was 8 years [17]. Byl found that after sensorimotor training there was 70-94% improvement in motor control in 8 of 12 patients with task-specific dystonia and adequate to return to their former occupation [75]. However the study used the unaffected hand as the control and did not have a separate control group. Another method, called pedagogical retraining, utilizes behavioral approaches and movement of the dystonic body part to a tempo that doesn’t induce dystonia. Subjects are given visual feedback with mirrors, monitors and taught body awareness techniques. The process took 24 months and 24% of subjects had improvement in motor control in a large series of 145 musicians [76]. The degree of improvement allowed them to return to a professional musician career. Another study found 50% of subjects had subjective improvement after an average of 28 months of training. In contrast, a small study of guitarists with musician’s dystonia using cathodal transcranial direct current stimulation failed to improve motor control [18].

Immobilization has been shown to reduce cortical representation of the immobilized body part in healthy subjects. Immobilization has had limited success with musician’s dystonia. In addition, studies contained small number of subjects without controls and were limited by observer bias. In one study, only 6 of 19 patient reported marked improvement after immobilization with a splint for 4 to 6 weeks. In one study, 63% of subjects reported subjective improvement after 35 months of ergonomic changes where the affected digit was immobilized with a splint to block dystonic movements [72].

## CONCLUSION

Musicians’ dystonia is increasingly recognized in the medical and musical community. Advances in neuroimaging and novel techniques have shed light on its underlying pathophysiology. To date, a deranged cortical plasticity leading to abnormal sensorimotor integration, combined with reduced inhibition across several levels of the motor pathway are likely mechanisms. Together with the understanding of the potential risk factors and development of targeted medical therapy and rehabilitation programs, the outlook is hopeful for real progress in the near future. 
